# Axial Ablation versus Terminal Interruption of the Reflux Source (AAVTIRS): A Randomised Controlled Trial

**DOI:** 10.1177/15385744241265750

**Published:** 2024-07-21

**Authors:** Keohane CR, Westby D, Twyford M, Aherne T, Tawfick W, Walsh SR

**Affiliations:** 1 8799 National University of Ireland Galway, Galway, Ireland; 2 University Hospital Galway, Galway, Ireland

**Keywords:** venous leg ulcers, terminal interruption of the reflux source, endovenous ablation

## Abstract

**Introduction:**

Treatment of reflux has been shown to improve time to healing of Venous Leg Ulcers (VLU). Terminal Interruption of the Reflux Source (TIRS) treats reflux within the plexus of veins around an active VLU using foam sclerotherapy. The efficacy of TIRS in managing VLU has never been tested.

**Methods:**

We performed a pragmatic, single centre, assessor-blinded, randomised controlled trial comparing endovenous ablation of the axial superficial veins (Axial Ablation-AA) vs TIRS. Patients of any age with VLU of any duration were eligible.

**Results:**

98 Participants were randomised to AA or TIRS. 39/55, 70.9% (95%CI; 57.1-82.37) healed their VLU in the AA group, while 29/39, 74.36% (95%CI; 57.87-86.96) healed their VLU in the TIRS group, *P* = 0.45.4 were lost to follow-up. Median time to ulcer healing was 84 days (95%CI; 74.67-93.33) in the axial ablation group and 84 days (95%CI; 73.02-94.98) in the TIRS group. Hazard Ratio for ulcer healing with AA vs TIRS was 0.96 (95%CI 0.59-1.56). There were no significant quality of life differences.

**Conclusion:**

The AAVTIRS trial did not show that axial ablation was superior to TIRS in the primary outcome of number of VLU healed in 6 months, or time to VLU healing. This trial is not powered to show non-inferiority. TIRS is a viable option for treatment of VLU. Further investigation is necessary before it can be recommended as an alternative to axial ablation.

Trial registered at clinicaltrials.gov NCT04484168.

## Background

Venous leg ulcers (VLU) are a massive burden on health services internationally.^1-4^ In Ireland, prevalence of leg ulcers is 1 per 800 people in the general population, rising to 1 per 100 over the age of 70 years.^1^ Compression therapy has been shown to expedite healing of VLU.^5,6^ However, it is time consuming for patients and practitioners and requires significant community support.

Surgical treatment of varicose veins, in combination with compression, has been shown to improve the rate of ulcer recurrence^7^ but the Effect of Surgery and Compression on Healing and Recurrence (ESCHAR) study didn’t show any benefit in ulcer healing from surgery.^7,8^ Numerous observational studies^9-12^ and in particular the Early Venous Reflux Ablation (EVRA) trial have since demonstrated that after endovenous treatment of reflux, the time to ulcer healing is reduced.^13^ This has important implications for the patient, their quality of life,^14^ and for the economic burden on community and hospital services of ongoing ulcer management.

Endovenous treatment of reflux can be achieved by ablation of the main superficial veins of the leg, or Axial Ablation, but there is also a growing body of evidence in favour of strategies which aim to preserve these axial veins, such as CHIVA (French Cure Conservatrice et Hémodynamique de l'Insuffisance Veineuse en Ambulatoire)^15^ and ASVAL (French Ambulatory Selective Variceal Ablation under Local anaesthetic.^16^ Terminal Interruption of the Reflux Source (TIRS) aims to directly target those veins in the peri-ulcer plexus in the leg which should normally drain the ulcerated area, and by only treating these specific veins, treat reflux locally. Numerous small studies have reported success in managing VLU with TIRS and healing ranging from 83% to 100%.^17,18^ TIRS has never before, to our knowledge, been tested in a randomised trial.

There are numerous means of achieving TIRS. One method that is commonly used is foam sclerotherapy, which can be ultrasound guided or directed by other means such as near infrared LASER vein viewer.^19^ This can be performed using a small needle, often without even local anaesthetic and can be delivered as an office-based treatment. This makes it an attractive method in terms of resource use and acceptability to patients, especially in our experience, the elderly. Foam sclerotherapy has been shown to be safe and effective, including perilesional sclerotherapy.^17,18,20^

We conducted the AAVTIRS trial to assess whether, in patients with VLU, TIRS is a viable alternative to axial ablation in achieving complete VLU healing.

## Methods

### Trial Design and Oversight

AAVTIRS was a prospective, single centre, parallel group, assessor-blinded randomised controlled trial. No funding was received and ethical approval was obtained from Galway University Hospitals Clinical Research Ethics Committee. All data was collected by the trial coordinator, under oversight from the department of vascular surgery at University Hospital Galway. Details of the trial design and implementation are provided in the protocol, which has been published previously.^21^ The trial was registered at clinicaltrials.gov, reference number NCT04484168.

### Setting and Participants

Participants were recruited at the Leg Ulcer Centre Ireland, operating out of Roscommon University Hospital. Patients were considered for enrolment if they had a primary or recurrent VLU, with great or small saphenous vein reflux confirmed on ultrasound assessment, and were suitable for compression therapy.

Reflux was defined as retrograde flow lasting for >0.5 seconds as measured on duplex in the standing position. Suitability for compression was defined as having palpable pedal pulses, or Ankle-Brachial pressure Index (ABI) ≥ 0.8, or Toe-Brachial Index (TBI) ≥ 0.5 and no history of hypersensitivity or other intolerance to compression bandages.

Patients were excluded if clinical assessment suggested a non-venous aetiology, if they had active or recent (within 2 weeks) infection of their ulcer, could not receive sotradecol (eg, pregnancy or breastfeeding, hypersensitivity to sotradecol), were unable to provide informed consent, had isolated perforator vein reflux only or had evidence of deep venous occlusion.

Patients already attending the leg ulcer clinic were excluded from enrolment with the same VLU but were eligible to enrol with a contralateral VLU.

### Sample Size

A prospective calculation of sample size^22^ was informed by existing evidence relating to AA,^9-11,13^ predominantly the EVRA trial,^13^ which showed an unadjusted rate of ulcer healing at 24 weeks of 85.6%. A more modest success rate was expected because exclusion criteria did not limit participants to new ulcers. 80% was felt to be reasonable. TIRS has very limited existing evidence, with reported healing rates ranging from 83% to 100%.^17,18^ While this suggested comparable outcomes, the evidence for TIRS to date consists of small case series and this was felt to be somewhat unreliable to inform a sufficiently robust power calculation. We elected to utilise an adaptive approach and commence recruitment with initial enrolment targets calculated for non-inferiority based on the available lower quality data but with a planned interim analysis after 50 patients had completed follow up to allow revision of recruitment targets to reflect a better informed power calculation. The trial would cease recruitment if 1 arm demonstrated statistically significant superiority at the interim analysis stage. In light of the acknowledged weaknesses in the initial non-inferiority power calculation, the trial could switch to a recruitment target based on a power calculation for superiority if the interim analysis revealed a trend in favour of 1 arm that wasn’t statistically significant but the interim power calculation indicated a revised recruitment target for non-inferiority that was not feasible.

A non-inferiority trial with an 80% success rate in both arms would necessitate a cohort of 308 patients to ensure adequate power at the 5% significance level. By the time of the interim analysis in August 2021 there was a notable trend in favour of 1 arm, which was not statistically significant but a non-inferiority trial was no longer feasible. A sample size calculation, based on superiority and the trend in the results to date indicated that 98 participants would be required to give 80% power to detect difference in the primary outcome at the 5% significance level in a superiority trial.

### Randomisation and Blinding

Once enrolled, patients were randomised on a 1:1 basis to axial ablation or TIRS with stratification by ulcer size (<5 cm^2^, 5.1 to 10 cm^2^, 10.1 to 25 cm^2^, >25 cm^2^). The randomization sequence was a computer generated random number sequence generated by the trial coordinator (CRK) with allocation concealment using sequentially marked sealed opaque envelopes. The surgical team performing the interventions were necessarily not blinded to allocation, nor were participants, due to the need to provide informed consent. Patients were first enrolled by a member of the trial team, who remained blind to allocation, and gave their consent to participate in the trial. The same trial team member then provided the next numbered envelope unopened to the surgical team. Once opened the allocated intervention was explained to the patient for them to consent to the intervention.

All members of the trial team not involved in the procedure remained blind to allocation, including trial assessors and the trial coordinator. No member of the surgical team who was unblinded had any interaction with the participants after their intervention until their VLU was fully healed, or they completed 6 months of follow up.

### Interventions

*TIRS.* TIRS was performed by first assessing the peri-ulcer plexus of veins to identify suitable injection sites to maximise treatment area with the fewest feasible punctures. A sclerosant foam was then created using Sotradecol (Sodium-Tetradecyl-Sulphate) agitated with air. A 1:4 sotradecol:air ratio was used, in 2 5 mL syringes in a modified Tessari method,^23^ creating 5 mL foam. This method was used for all patients receiving TIRS, with up to an additional 5 mL foam allowable to ensure satisfactory spread of foam throughout the target veins.

*Axial Ablation.* Final choice of the method of ablation was at the discretion of the treating surgeon. Mechanical Occlusion with Chemical Assistance (MOCA) was the preferred method as it is a non-thermal technique, and so could be used in more superficial veins than thermal techniques and this was expected to facilitate use in a wider range of anatomies. Clarivein^TM^ (Vascular Insights LLC, Quincy, USA) uses a catheter to inject sclerosant alongside a rapidly rotating wire. The wire scores the endothelium of the vein as well as spreading the sclerosant around the full circumference of the vein, augmenting the inflammatory response.

For both arms of the study compression with Coban^TM^ (3M, Minnesota, USA) was commenced immediately upon completion of the procedure. A pragmatic approach was used regarding frequency of dressing changes and choice of primary dressings. Choice of initial primary dressing was at the discretion of the trial nurses. Dressing changes were recommended twice weekly initially and at least once weekly for the duration of follow up. Outside of trial follow-up visits, dressing changes were done locally by patients’ public health or general practice nurse. Consultation with the trial nurses was encouraged, to try to maintain a degree of homogeneity, but ultimately these local services were not given a prescribed plan beyond their own experience and training.

### Outcomes and Assessment

The primary outcome measure was the number of VLU which healed within 6 months of randomisation, with healing defined as complete re-epithelialisation of the index limb.

Secondary outcomes included time to ulcer healing; wound regeneration as measured using the Bates-Jensen Wound Assessment Tool (BWAT)^24^ at each monthly follow up visit; change in VLU area, estimated with a grid and categorised using the same scale as the BWAT (<4 cm,^2^ 4-16 cm^2^, 16.1-36 cm^2^, 36.1-80 cm^2^, >80 cm^2^); venous disease severity using the Venous Clinical Severity Score^25^ at monthly follow up, and ulcer-related quality of life as assessed using the Charing Cross Venous Ulcer Questionnaire at enrolment and upon exiting the trial by healing or reaching 6 months of follow-up.^26^

*Assessment.* Trial assessors were qualified tissue viability nurses who screened all leg ulcers attending the clinic for suitability. On enrolment, baseline VCSS and CCVUQ were completed by the patient and the trial assessors documented baseline BWAT score.

Participants were invited to monthly follow-up visits, at which BWAT and VCSS were documented. Trial assessors determined when a wound was healed. Upon healing, or having completed 6 months of follow up, the participant exited the trial, and a final CCVUQ was again documented.

### Statistical Analysis

Statistical analysis was performed using StatsDirect Statistical Package version 3 (StatsDirect Ltd, Cambridge, UK.). All analyses were performed on an intention to treat basis and

Continuous variables are presented as mean ± standard deviation (SD) or median ± interquartile range (IQR) based on parametric or nonparametric distribution. Categorical and ordinal variables are described in absolute numbers with percentage frequencies. For the primary outcome, Fisher’s exact test was used to test for significance due to the overall small sample size. Logistic regression was used to evaluate the impact of potential confounding variables on this dichotomous outcome. Kaplan-Meier time to event analysis with log-rank test were used to evaluate time to ulcer healing. The Mann-Wittney U and Wilcoxon Signed-Rank tests were used to assess paired and unpaired non-parametric data such as quality of life outcomes, BWAT scores and ulcer area.

## Results

Patients were enrolled from July 2020 through April 2022, during which time 585 consecutive patients with leg ulcers were screened, and 99 eligible VLU consented to participate. The main reason for non-enrolment were peripheral vascular disease leading to exclusion, or refused consent. One patient healed between enrolment and randomisation so was therefore not randomised. Baseline characteristics of the 98 randomised participants are shown in table 1.

**Table 1.** Baseline Characteristics Age and Ulcer Size Are Represented In Median (IQR) BMI as mean (95%CI), all others as frequency (%)TIRS = terminal interruption of the reflux source, BMI = body Mass index, DVT = deep vein thrombosis, BWAT = bates-jensen wound assessment, VCSS = venous clinical severity score, CCVUQ = charing cross venous ulcer questionnaire.

Characteristic		Axial Ablation	TIRS
Age		73 (60-79)	67 (60-78)
BMI		30.36 (27.78-32.93)	31.77 (29.1-34.47)
		N (%)	N (%)
Gender	Female	36 (65.5)	19 (65.5)
	Male	19 (34.5)	10 (34.5)
Smoking	Current	4 (7.5)	2 (5.4)
	Former	17 (32.1)	10 (27.03)
	Never	32 (60.4)	25 (67.57)
	Not recorded	2	2
Obesity	Normal	6 (10.9)	5 (12.82)
	Overweight	17 (30.9)	8 (20.51)
	Obese	15 (27.27)	15 (38.46)
	Morbidly obese	4 (7.27)	2 (5.13)
	No data	13 (	9 (
Diabetes		12 (22.64)	7 (18.92)
Immunosuppression		4 (7.5)	4 (10.5)
History of ipsilateral DVT		1 (1.89)	0
History of ipsilateral ulcer		21 (42.86)	17 (47.22)
Ulcer duration (months)		6 (2-12)	5 (2-9)
Ulcer size	<5 cm^2^	19 (34.54)	17 (43.59
	5-10 cm^2^	14 (25.45)	10 (25.64)
	10-25 cm^2^	7 (12.72)	4 (10.25)
	>25 cm^2^	15 (27.27)	8 (20.51)
		Median (IQR)	Median (IQR)
Ulcer size		8.04 (3.6-39.58)	5.96 (2.87-12.84)
BWAT at enrolment		28.55 (26.85-30.24)	27.23 (25.04-29.43)
VCSS at enrolment		13 (11-15)	12 (10-15)
CCVUQ at enrolment		51 (36-60)	55 (39-62)

There were no significant differences in the demographic makeup of the 2 arms in the trial. There were no significant differences in any documented baseline risk factors for impaired wound healing such as smoking, diabetes and immunosuppression between the axial ablation and TIRS groups. More VLU specific factors such as duration, size, and history of DVT were also not significantly different. Median age in the Axial Ablation group was higher but this was not statistically significant (*P* = 0.17). Patients were followed up until their ulcer healed, or they had completed 6 months of follow up post-intervention with the last patient randomised in April 2022 and follow up completed in October 2022.

During follow up COVID-19 restrictions interfered significantly with attendance at the clinic. Enforced reductions in the number of ‘non-urgent’ patients allowed to attend clinics and the need to balance the competing interests of trial follow up with prioritisation of clinically urgent cases meant that some patients did not attend for assessment by the designated trial assessors. This primarily affected assessment of secondary outcomes and quality of life outcomes in particular, while the primary outcome in some cases was confirmed by phone call to the patient’s primary care doctor or primary care nurse. Reduced clinic visits and staffing did not allow an opportunity to re-scan after intervention to document whether ablation had been successful. Four patients withdrew consent or were considered lost to follow up as the primary outcome could not be confirmed.

95 (96.94%) interventions were performed within 2 weeks of randomisation, 1 intervention happened within 3 weeks and the remaining 2 within 5 weeks. All participants underwent their assigned intervention. Two patients in the ablation group underwent repeat ablation before completing follow-up due to persistent reflux with poor progress with their VLU.

### Primary Outcome

Within 6 months of treatment, 39 of 55 (70.9%) VLU in the axial ablation group had fully healed, while 29 of 39 (74.36%) VLU in the TIRS cohort had fully healed. This was not statistically significant (unadjusted odds ratio (OR) = 0.84; 95%CI 0.295 to 2.31; *P* = 0.449). Logistic regression did not indicate a significant impact on this outcome for any of the following variables: age, current smoking, diabetes, obesity, ulcer size, ulcer duration and history of previous ipsilateral VLU.

### Secondary Outcomes

**Figure 1.**
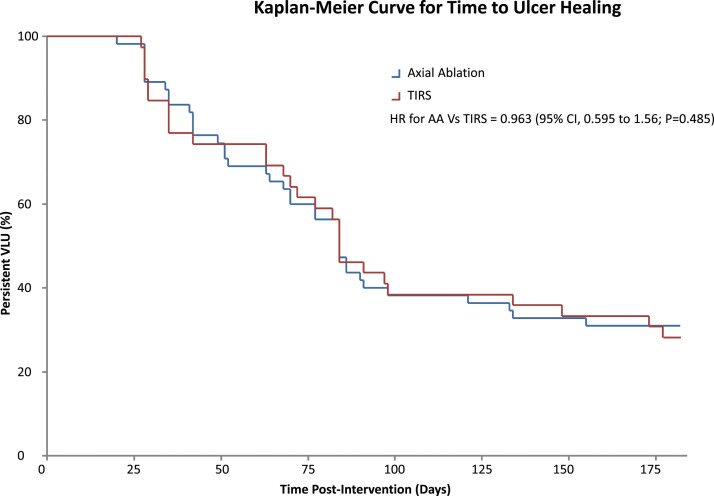
Kaplan-Meier Curve for Time to Ulcer Healing X-axis = Percentage of VLU remaining unhealed Y-axis = Time since intervention (censored at 6 months); HR= Hazard Ratio AA = Axial Ablation, TIRS = Terminal Interruption of the Reflux Source.

There was similarly no significant difference in time to ulcer healing. Among those who had a confirmed date when their VLU healed, median time to healing was 68 days (IQR, 41 to 86) in the axial ablation group and 72 days (IQR, 35 to 91) in the TIRS group. Kaplan-Meier time to event analysis was used to demonstrate cumulative hazard (Figure 1), with censorship of non-healed VLU at 182 days and on this basis median time to ulcer healing was similar in both groups; Axial Ablation 84 days (95% CI 74.67 to 93.33) vs TIRS 84 days (95%CI 73.016 to 94.983). Hazard ratio = 0.963 for Axial Ablation vs TIRS (95% CI, 0.595 to 1.56; *P* = 0.485).

The BWAT was used as both an objective method to assess for a baseline difference in VLU severity, as well as providing a less simplistic impression than size alone to indicate if any significant difference in VLU improvement existed between the groups, especially among those VLU that did not fully heal. BWAT scores improved significantly in both arms, median improvement of 18.5 (IQR, 16 to 21) points with Axial Ablation and 15.5 (IQR, 12 to 18.5) with TIRS, *P* < 0.001 for each. There was no significant between-group difference in BWAT score *P* = 0.322 overall, or among those whose VLU did not heal *P* = 0.206.

Finally, there was there was no significant reduction in VLU size in either group among those whose VLU did not heal; Axial Ablation, *P* = 0.5, TIRS, *P* = 0.375.

### Quality of Life

Quality-of-life outcomes are summarized in table 2. At baseline, mean CCVUQ scores did not differ significantly between the treatment arms, Axial Ablation 51 (IQR, 36-60) vs TIRS 55 (IQR, 39-62) *P* = 0.567. Likewise VCSS was similar in both treatment arms; Axial Ablation 13 (IQR, 11-15) vs TIRS 12 (IQR, 10-15), *P* = 0.584.

**Table 2.** Quality of Life ScoresVCSS= Venous Clinical Severity Score, CCVUQ= Charing Cross Venous Ulcer Questionnaire IQR= Inter-Quartile Range.

Outcome		Valid n	Median (IQR)	Valid n	Median (IQR)
VCSS					
	At enrolment	54	13 (11-15)	39	12 (10-15)
	1 Month	35	8 (6-13)	31	10 (7-14)
	2 Months	18	9 (6-12)	21	11 (7-12)
	3 Month	17	11 (5-14)	14	9 (6-13)
	4 Months	6	10.5 (10-14)	5	10 (9-11)
	5 Months	6	12 (11-14)	5	11 (10-16)
	At completion	36	6 (5-7.5)	28	5.5 (4-8.5)
CCVUQ					
	At enrolment	45	51 (36-60)	30	55 (39-62)
	At completion	33	31 (23-48)	24	35.5 (25-51)

Both groups had a significant overall reduction in CCVUQ and VCSS, ie, an improvement in quality of life. Median improvement in CCVUQ in the Axial Ablation group was 11 (0.5 to 22), *P* = 0.042, and 11.5 (3 to 22), *P* = 0.009 in the TIRS group. Median improvement in VCSS in both groups at completion was 6.5 (5 to 8), *P* < 0.001. VCSS was assessed monthly, with no significant between-group difference at any stage during follow up, and indeed both groups followed a similar pattern with an initial reduction in VCSS from enrolment to first follow-up, with a late return towards baseline VCSS from 4 months.

At completion (ie, healing or completion of 6 months of follow up) there was no statistically significant between-group difference in either CCVUQ, 31 (23-48) vs 35.5 (25-51), *P* = 0.497 or VCSS6 (IQR, 5-7.5) vs 5.5 (IQR, 4-8.5), *P* = 0.753.

While quality of life improved for both treatment groups overall, these results were not shared by the cohort in whom VLU did not heal, with no significant improvement in VCSS or CCVUQ in either group; for VCSS, *P* = 0.5 with Axial Ablation and *P* = 0.25 with TIRS, and for CCVUQ, *P* = 0.203 with Axial Ablation and *P* = 0.812 with TIRS. Once again there were no significant between-group differences in VCSS, *P* = 0.584, or CCVUQ, *P* = 0.453.

There were no ulcer recurrences documented by 6 months.

## Discussion

This single centre randomised trial found no significant difference, over up to 6 months of follow-up, in rates of venous leg ulcer healing between those treated with ablation of reflux in the saphenous veins compared with those treated with terminal interruption of the reflux source using foam sclerotherapy. Patients reported a significant improvement in their ulcer specific and venous disease specific quality of life with both treatments, without a significant difference between treatment groups. This quality of life benefit was not shared by participants whose VLU did not heal.

Two large randomised trials have demonstrated the benefits of treating superficial venous reflux in patients with VLU. The ESCHAR study showed that superficial venous surgery plus compression reduced the rate of ulcer recurrence compared to compression alone, but did not show a significant difference in healing, while the EVRA trial showed that early endovenous ablation of reflux improved time to ulcer healing and healing rates at 6 months.^13^

Since the publication of the EVRA trial we have endeavoured at our centre, to offer reflux ablation where feasible. The typical patient presenting with VLU at our centre is elderly with multiple comorbidities and often, when offered ablation of superficial venous reflux, intervention has been declined. This provides some explanation for the overall trial recruitment rate of about 17% (99 of 585 patients screened). Many patients do not want ‘an operation’, but are more than happy to have foam injections. While the EVRA trial showed that early reflux ablation is effective in reducing the time to ulcer healing, there has been a lack of quality evidence to justify the use of TIRS. This led us to conduct this trial to better understand whether TIRS would achieve similar benefits.

AAVTIRS was planned to be adaptive because of the paucity of evidence underpinning the initial power calculation. The evidence around TIRS was considered optimistic at best, and so in the absence of better evidence the decision was taken to base the initial recruitment target on these studies which indicated a non-inferiority trial with an assumption of 80% healing in both trials arms with a 10% non-inferiority limit was reasonable. An interim analysis was planned after the first 50 patients completed follow up to allow for a more robust power calculation. Fairly standard limits for estimating sample size at the outset of trials, 80% power at the 5% significance level, were utilised as it was accepted from the outset that because the original power calculation was likely flawed, this interim calculation would represent the first robust power calculation. It was also accepted that because the initial recruitment target for non-inferiority was based on optimistic and probably quite selective series, the change from non-inferiority to a superiority target might be necessary. At the interim analysis 96.4% (27/28) had healed in the Axial Ablation arm compared to 78.26% (18/23) in the TIRS arm. As outlined above, the sample size was recalculated, suggesting that 98 patients would provide sufficient power to detect significant superiority with 80% power at the 5% significance level. The trial was, therefore, continued until 99 patients had been recruited. The actual healing rates observed (70 to 75%) were lower than originally assumed or suggested by the interim power calculation. Assuming a healing rate of 70% in each arm and a 5% non-inferiority limit, a further trial would require 1040 patients in each arm (2080 patients plus 10% for withdrawals and dropouts). Based upon the observed recruitment rate in AAVTIRS, achieving this sample would require screening approximately 12000 patients. Healing rates in the AA arm fell dramatically after the interim analysis from 96.4% to an overall healing rate of 70.9%. Nothing in our analysis accounts for this discrepancy. Healing rates in our trial are less than that seen in either arm of the EVRA trial. We attribute this to the chronicity of many of the ulcers in our cohort. An important and often overlooked element of the EVRA trial is its focus on early intervention. The excellent healing rates seen in the compression-only group in particular suggest that early high-quality compression can go a long way to achieving exceptional healing results. The decision was taken at the outset of the AAVTIRS trial not to restrict eligibility based on VLU chronicity on a very practical basis. Prior experience within the unit was that the majority of patients were not referred until their wound was persistent for a few months at least. This decision was later justified by the fact that even with prioritised appointments and same-day assessment and treatment, median ulcer duration was 5.5 months at enrolment.

Routine duplex assessment after intervention was not performed. While useful information can be gathered from technical success rates, the outcome of concern was VLU healing. There were also more practical reasons. The TIRS cohort would all be expected to have ongoing proximal reflux, difficult to distinguish from failed AA. As the accepted standard treatment, AA would be indicated for patients found to have ongoing reflux unless staff performing duplex knew the treatment groups in advance, otherwise mislabelling of TIRS as failed AA could have caused inordinate numbers of crossovers. In the context of the COVID-19 pandemic, only blinded clinic staff (assessors and trial coordinator) met with patients during follow up to minimise healthcare contacts and these staff could not assess for failed ablation while remaining blinded. Hospital based duplex scans were not feasible during Covid restrictions when only clinically essential hospital attendances could be justified.

No VLU recurrences were noted during follow-up of this cohort. All participants were asked, and many have given their consent to be contacted for longer term follow up in future to look at recurrence rates.

The VCSS is an objective measure of the severity of venous disease, encompassing both ulceration and wound factors, as well as non-wound related signs and symptoms^25^ while the CCVUQ is a quality of life questionnaire specific to venous leg ulcers.^26^ Both of these were improved significantly in both groups, but not among those whose VLU did not heal. This seems to suggest that in this cohort the benefits are derived predominantly from healing, while treatment of great and/or small saphenous venous reflux does not confer a significant benefit in and of itself. Low numbers in the non-healing cohort however make it difficult to draw any strong statistical inference.

### Strengths and Limitations

Several difficulties were encountered in the running of this trial. Recruitment was slower than anticipated and efforts to gather robust secondary outcome data were hampered by reductions in the number of patients attending all outpatient clinics due to COVID-19 restrictions combined with a nationwide health service cyberattack and data loss in 2021. The primary outcome could be affected by having been obtained through phone calls in some instances. Patient reported outcomes can be associated with more false positives. Despite planned 1:1 randomisation, the 2 treatment groups were unbalanced. Block randomisation was not employed. There is some risk of selection bias given the relatively large number of patients screened, with only 17% of screened patients being enrolled. The majority of these were patients with arterial or mixed ulcers unsuitable for compression.

The trial does have some strengths. Ulcer duration was not an exclusion criterion, resulting in a trial population more representative of daily practice in many centres, particularly in rural settings. Assessor blinding of the outcome measures removes the possibility of observer bias influencing the results. To date, it provides the only randomised, efficacy data comparing ulcer outcomes in patients receiving axial ablation vs TIRS.

## Conclusion

In conclusion this assessor-blinded randomised controlled trial did not show ablation of superficial venous reflux in the saphenous veins to be superior to foam sclerotherapy, injected into the peri-ulcer venous plexus, in the treatment of VLU as there was no significant difference in rates of healing at 6 months.

## Data Availability

The datasets during and/or analysed during the current study available from the corresponding author on reasonable request.*
